# Patients With Ischemic Core ≥70 ml Within 6 h of Symptom Onset May Still Benefit From Endovascular Treatment

**DOI:** 10.3389/fneur.2018.00933

**Published:** 2018-11-05

**Authors:** Zhicai Chen, Ruiting Zhang, Ying Zhou, Xiaoxian Gong, Meixia Zhang, Feina Shi, Xinfeng Yu, Min Lou

**Affiliations:** ^1^Department of Neurology, The Second Affiliated Hospital of Zhejiang University, School of Medicine, Hangzhou, China; ^2^Department of Radiology, The Second Affiliated Hospital of Zhejiang University, School of Medicine, Hangzhou, China

**Keywords:** acute ischemic stroke, large core, endovascular treatment, intravenous thrombolysis, reperfusion, outcome

## Abstract

**Background:** Large core is associated with poor outcome in acute ischemic stroke (AIS) patients. It is unclear whether endovascular treatment (EVT) could bring benefits to patients with core volume ≥70 ml before treatment. We aimed to compare the impact of EVT with intravenous thrombolysis (IVT) on the outcome in patients with core volume ≥70 ml.

**Methods:** We included consecutive anterior circulation AIS patients who underwent MR or CT perfusion within 6 h post stroke onset, which revealed a core ≥70 ml before reperfusion therapy. Good outcome was defined by modified Rankin Scale of 0 to 2 at 90-day. Reperfusion was defined as a reduction in hypoperfusion volume of ≥70% between baseline and 24 h.

**Results:** One hundred four patients were included. Among them, 76 received IVT only, and 28 received EVT. After adjusting for age, NIHSS score, baseline core volume and onset to imaging time, patients in EVT group were more likely to achieve good outcome compared to IVT patients (OR, 3.875; 95% Cl 1.068–14.055, *p* = 0.039). More patients in EVT group achieved recanalization (84.0 vs. 58.5%, *p* = 0.027) and reperfusion (66.7 vs. 33.3%, *p* = 0.010) than in IVT group. Reperfusion also independently predicted good outcome (OR, 7.718; 95% Cl 1.713-34.772, *p* = 0.008). All patients with good outcome achieved recanalization at 24 h.

**Conclusions:** Our data indicated that patients with core volume ≥70 ml might still benefit from EVT, which was related to its high reperfusion rate.

## Introduction

Previous studies have demonstrated that acute ischemic stroke (AIS) patients with large core, usually ≥70 ml, had low rate of favorable outcome, ranging from 0 to 21% (1–4). Endovascular therapy, which has been proved to be the most effective way in acute phase of stroke with large artery occlusion, was considered futile for patients with large core. Thus most endovascular treatment (EVT) trials have excluded these patients. For instance, in the Diffusion Weighted Imaging Evaluation for Understanding Stroke Evolution Study-2 (DEFUSE-2) (5) and Extending the Time for Thrombolysis in Emergency Neurological Defcits-IntraArterial (EXTEND-IA) trial (6), core ≥70 ml, was an exclusion criterion. However, this conception came from the research conducted in patients who received intravenous thrombolysis (IVT) that the malignant profile could be associated with higher rate of symptomatic intracerebral hemorrhage (sICH) poor outcome (1, 7).

Despite of large core, hypoperfusion tissue would still have chance to be salvaged from reperfusion (8). While EVT is significantly related to high rate of recanalization and reperfusion (9), the idea that patients with large core should be excluded from EVT has been challenged recently. Gilgen et al. found that in patients treated with EVT, favorable outcome was achieved after successful reperfusion in every third patient with DWI lesions >70 ml, whereas after poor or failed reperfusion, outcome was favorable in only every 12th patient (10). Similarly, Olivot et al. also observed that in EVT patients, reperfusers tended to achieve favorable outcome compared to non-reperfusers (42.9 vs. 8.3%) (4). These promising results brought out the question whether EVT would bring relatively better outcome for patients with infarct lesion >70 ml, compared to IVT, in view of the increased rate of reperfusion by EVT. Indeed, to our knowledge, the efficacy and safety of EVT in patients with core ≥70 ml has not yet been evaluated, as directly compared to IVT.

We aim to evaluate the benefit and risk of EVT in patients with baseline core ≥ 70 ml, in contrast to IVT, and further assess the impact of recanalization and reperfusion on clinical outcome in the same population.

## Materials and methods

### Patients

We retrospectively reviewed the consecutive clinical and imaging data of AIS patients who received reperfusion therapy within 6 h of symptom onset between 2010 and 2017. We included patients who (1) had a diagnosis of anterior circulation AIS confirmed by DWI or CT perfusion (CTP); (2) underwent magnetic resonance perfusion (MRP) or CTP and had a core ≥70 ml at baseline (3) had complete follow-up at 3 months.

### Ethics statement

All subjects had given written informed consent prior to the study, and the protocols had been approved by the human ethics committee of the second affiliated hospital of Zhejiang University, School of Medicine. Clinical investigation had been conducted according to the principles expressed in the Declaration of Helsinki.

### Study treatments

Before 2013.9, all patients were treated with IVT. From 2013.10, eligible patients were treated with IVT and EVT according to local guidelines, the clinical judgment of the treating physician and the preferences of patients or patients' relatives. In clinical practice, for patients with core>70 ml, neurologist would elaborate the patients' situation and risks of different treatments in detail. Neurologists would emphasize that EVT might bring benefits for the patient according to our experience but still lack of evidence and the support of authoritative guidelines. If the patients' family members could understand the situation and agreed to the treatment, we would perform EVT with informed consent. Besides, considering that EVT is much more expensive than IVT, patients who underwent EVT may have a more wealthy family than patients who underwent IVT. Finally, patients who underwent EVT tended to be younger than patients who underwent IVT since family members would find it difficult to accept the death of young patients. For IVT, patients received alteplase at a dose of 0.9 mg per kilogram (maximum dose = 90 mg). EVT group included patients who received only mechanical thrombectomy with the use of the second generation devices for thrombectomy, or bridging therapy, i.e., IVT before thrombectomy. Detailed procedure was described as in EXTEND-IA trial (6).

### Multimodal CT and MR protocol

CTP was performed on a 64-slice CT scanner (SOMATOM Definition Flash; Siemens Healthcare Sector, Forchheim, Germany), including NCCT scan (120 kV, 320 mA, contiguous 5 mm axial slices, acquisition time 7 s), and volume CTP (100 mm in the z-axis, 4 s delay after start of contrast medium injection, 74.5 s total imaging duration, 80 kV, 120 mA, slice thickness 1.5 mm, collimation 32 × 1.2 mm). Volume CTP consisted of 26 consecutive spiral acquisitions of the brain. All 26 scans were divided into 4 parts: (1) 2 scans with 3 s cycle time; (2) 15 scans with 1.5 s cycle time; (3) 4 scans with 3 s cycle time; and (4) 5 scans with 6 s cycle time. Axial slice coverage was 150 mm. A 60- ml bolus of contrast medium (Iopamidol; Braccosine, Shanghai, China) with a single injection was used at a flow rate of 6 ml /s, followed by a 20 ml saline chaser at 6 ml /s.

Subjects underwent MR on a 3.0T system (Signa Excite HD, General Electric Medical System, WI, USA). The MR protocol included an axial isotropic diffusion-weighted echo-planar spin-echo sequence and bolus-tracking perfusion weighted image (PWI). DWI was performed with a spin echo-planar sequence (field of view = 240 mm, slice thickness = 5 mm, number of slices = 18, slice gap = 1 mm, acquisition matrix = 160 × 160). PWI was performed with gradient echo-planar imaging (field of view = 240 mm, repetition time = 1,500 ms, echo time = 30 ms, acquisition matrix = 128 × 128. Total repetitions = 50, gadolinium dose = 15 ml, injection speed = 4–5 ml /s, scan duration = 1 min 15 s). The three-dimensional multi-echo SWI sequence used 11 equally spaced echoes: TE = 4.5 ms (first echo); inter-echo spacing 4.5 ms; TR = 58 ms; flip angle 20°; slice thickness 2.0 mm with no gap between slices. Magnitude images were used in further analysis.

### Image analysis

All perfusion images and apparent diffusion coefficient (ADC) images were retrospectively post-processed on commercial software (MIStar; Apollo Medical Imaging Technology, Melbourne, Australia). The time to maximum of tissue residue function (Tmax) map was produced using standard singular value deconvolution without arterial input function (AIF) delay. The AIF was automatically selected, which should be a global AIF from normal artery. Previously validated thresholds were applied in order to measure the baseline volume of hypoperfusion lesion (Tmax >6 s) and infarct core (relative cerebral blood flow (rCBF) < 30% on CT or ADC < 620 × 10^−6^ mm^2^/s on DWI), and 24 h infarct volume was measured on DWI or NCCT.

### Outcome measures

We used the Arterial Occlusive Lesion scale (grade 0: complete occlusion of the target artery; grade 1: incomplete occlusion or partial local recanalization at the target artery with no distal flow; grade 2: incomplete occlusion or partial local recanalization at the target artery with any distal flow; and grade 3: complete recanalization and restoration of the target artery with any distal flow) to define recanalization or no recanalization based on the presence (grade 2 or 3) or absence (grade 0 or 1) of any downstream flow. Reperfusion rate was defined as: (baseline hypoperfusion volume −24 h hypoperfusion volume)/baseline hypoperfusion volume, and reperfusion was defined as reperfusion rate ≥ 70% with baseline hypoperfusion volume ≥ 10 ml.

Clinical outcome at 3 months was assessed with modified Rankin Score (mRS) and dichotomized into good outcome (0–2) and poor outcome (3–6). Hemorrhage transformation (HT) and sICH were defined according to European Cooperative Acute Stroke Study (ECASS) II trial (11). Brain edema was assessed by a stroke neurologist (RZ) and a neuroradiologist (XY) independently by grading hemispheric swelling on a 7-point scale both at baseline and 24 h NCCT or DWI, and rater discrepancies were settled by consensus discussion (12). The degree of brain edema expansion was defined as the increase in grade from baseline to 24 h after treatment.

### Statistical analysis

The patients were dichotomised according to different treatments or clinical outcome. Fisher's exact test was used to compare the dichotomous variables between groups, while an independent sample two-tailed *t*-test or a Mann-Whitney *U*-test was used for the continuous variables, depending on the normality of the distribution. Age, baseline core volume, and variables with a *p* < 0.1 in univariate regression analyses were included in the binary logistic regression model. All analyses were performed blinded to the participant identifying information. Statistical significance was set at a probability value of < 0.05. All statistical analysis was performed with an SPSS package (V.14.0 for Windows).

## Results

**Figure 1:**
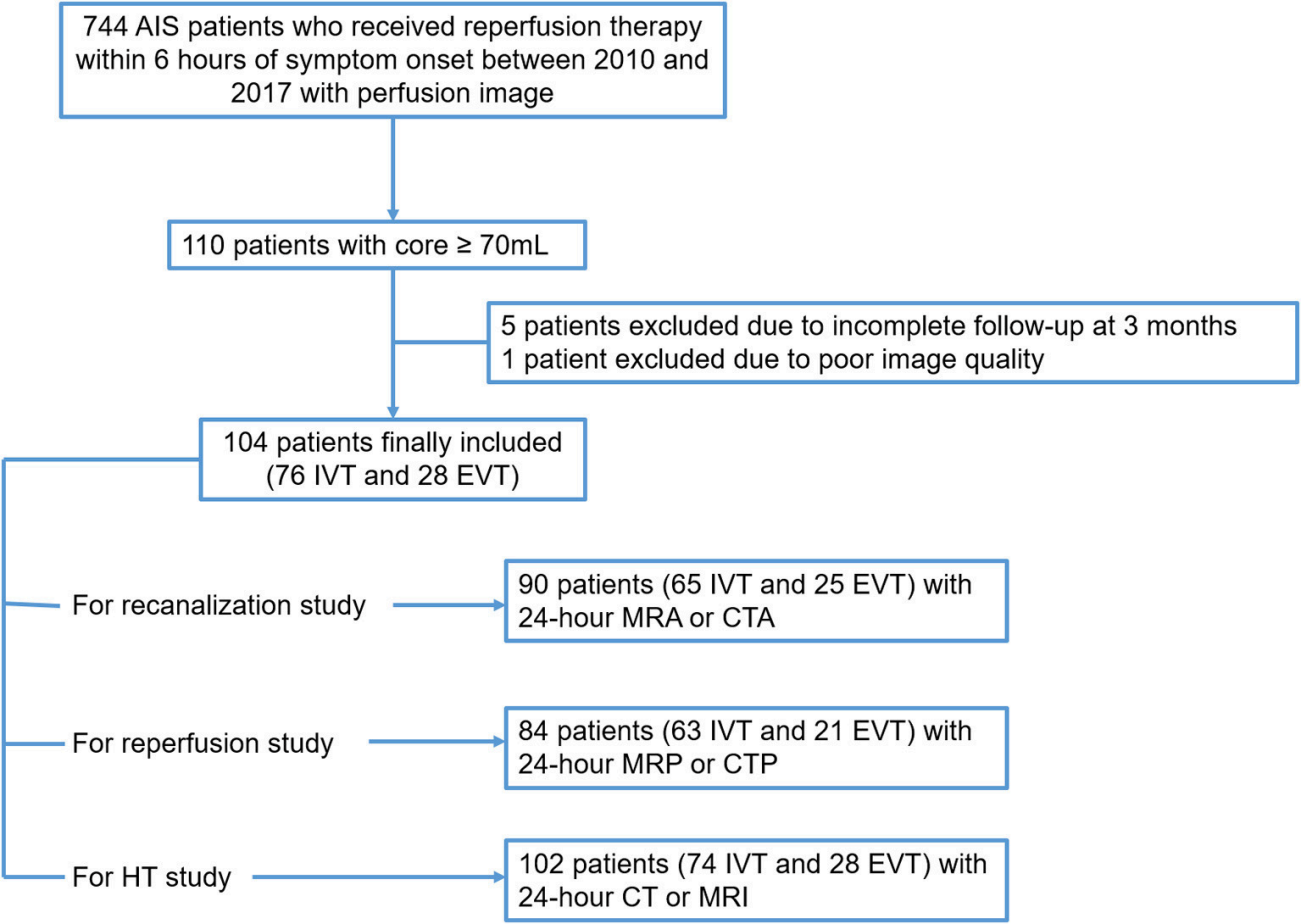
Patient flow chart. AIS, acute ischemic stroke; IVT, intravenous thrombolysis; EVT, endovascular treatment; HT, hemorrhage transformation.

Initially 744 AIS patients who received reperfusion therapy within 6 h of symptom onset between 2010 and 2017 with perfusion image were examined. 110 patients with core ≥70 ml at baseline were identified, 5 were excluded due to incomplete follow-up at 3 months. Another 1 patient was excluded due to poor image quality. Finally, 104 subjects were included in the analysis. Please see the flow chart (Figure 1).

For the 104 subjects [76 (73.1%) IVT and 28 (26.9%) EVT], the average age was 72.3 ± 12.6 and 32 (30.8%) were female. At baseline, 91 (87.5%) patients had CTP and 13(12.5%) had MRP. Median baseline NIHSS score was 16 (interquartile range, 13–19), 16 (15.4%) had 90-day a mRS score of 0–2 and 25 (24.0%) patients had 90-day a mRS score of 0–3.

Recanalization was analyzed in 90 patients [65 (72.2%) IVT and 25 (27.8%) EVT] who had 24 h MRA or CTA, and 59 (65.6%) patients achieved recanalization. Reperfusion was analyzed in 84 patients [63 (75%) IVT and 21 (25%) EVT] who had 24 h MRP or CTP, and 35 (41.7%) patients achieved reperfusion. HT was analyzed in 102 patients [74 (72.5%) IVT and 28 (27.5%) EVT] with 24 h CT or MRI, and 58 (56.9%) patients developed HT and 10 (9.8%) patients developed sICH.

As Table 1 shows, patients in EVT group and IVT group had comparable age, baseline NIHSS and baseline core volume. The percentage of patients who had baseline CTP for calculation of core volume was similar among 2 groups. More patients in EVT group achieved mRS 0–2 (32.1 vs. 9.2%, *p* = 0.011) and mRS 0–3 (39.3 vs. 18.4%, *p* = 0.038) at 90-day, compared with untreated IVT group.

**Table 1:** Clinical and imaging characteristics of patients who underwent different therapies.

	**IVT (*n =* 76)**	**EVT (*n* = 28)**	***p*-value**
Age, y	73.8 ± 11.8	68.4 ± 14.0	0.053
Female	26 (34.2)	6 (21.4)	0.240
**COMORBID CONDITIONS**			
Atrial fibrillation	41 (53.9)	16 (57.1)	0.827
Hypertension	57 (75.0)	19 (67.9)	0.466
Diabetes	15 (19.7)	5 (17.9)	1.000
Hyperlipidemia	21 (27.6)	8 (28.6)	1.000
Smoking	25 (32.9)	9 (32.1)	1.000
NIHSS score	16 (13–19)	16 (12–18)	0.422
Onset to imaging time, min	185 (112–231)	156 (95–224)	0.480
**RADIOLOGICAL DATA**			
Underwent CT at baseline	64 (84.2)	27 (96.4)	0.177
Baseline core volume, ml	114.6 (85.4–143.9)	97.8 (80.8–115.7)	0.059
Baseline hypoperfusion volume, ml	195.5 (145.1–255.2)	183.9 (150.6–211.3)	0.253
Baseline brain edema	0 (0–1)	1 (0–1)	0.385
Site of vessel occlusion
ICA	31 (40.8)	14 (50.0)	0.504
MCA	45 (59.2)	14 (50.0)
Underwent CT at 24 h^*^	40 (54.1)	19 (67.9)	0.263
24 h infarct volume, ml^*^	150.5 (80.8–248.5)	89.7 (30.0–185.7)	0.018
24 h brain edema^*^	3 (1–5)	3 (1–3)	0.157
Brain edema expansion within 24 h^*^	2 (1–4)	2 (1–2)	0.028
Recanalization^**^	38 (58.5)	21 (84.0)	0.027
Reperfusion^#^	21 (33.3)	14 (66.7)	0.010
HT^*^	37 (50.0)	21 (75.0)	0.026
sICH^*^	7 (9,5)	3 (10.7)	1.000
mRS < 3	7 (9.2)	9 (32.1)	0.011
mRS < 4	14 (18.4)	11 (39.3)	0.038
Death	26 (34.2)	7 (21.2)	0.478

* *Analyzed in 102 patients (74 underwent IVT and 28 underwent EVT)*.

** *Analyzed in 90 patients (65 underwent IVT and 25 underwent EVT)*.

# *Analyzed in 84 patients (63 underwent IVT and 21 underwent EVT)*.

*IVT, intravenous thrombolysis; EVT, endovascular treatment; NIHSS, National institutes of Health Stroke Scale; CTP, CT perfusion; HT, hemorrhagic transformation; sICH, symptomatic intracranial hemorrhage; mRS, modified Rankin Scale*.

More patients in EVT group achieved recanalization (84.0 vs. 58.5%, *p* = 0.027), reperfusion (66.7% vs. 33.3, *p* = 0.010), and developed HT (75.0 vs. 50.0%, *p* = 0.026) compared with IVT group, but sICH rate were comparable in these two groups. At 24 h, patients in EVT group had smaller infarct volume [89.7 (30.1–185.7) ml vs. 150.5 (80.8-248.5) ml, *p* = 0.018], and lower degree of brain edema expansion [2 (1–2) vs. 2 (1–4), *p* = 0.028] compared with IVT group.

**Figure 2:**
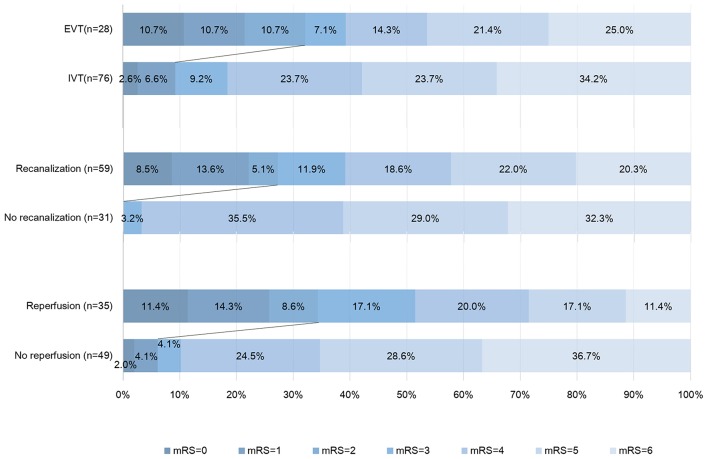
Distribution of modified Rankin Scale score (mRS) at 90 days. This figure showed the 90-day scores on the modified Rankin scale for the patients in different groups. Scores range from 0 to 6, with 0 indicating no symptoms, 1 no clinically significant disability (able to carry out all usual activities, despite some symptoms), 2 slight disability (able to look after own affairs without assistance but unable to carry out all previous activities), 3 moderate disability (requires some help but able to walk unassisted), 4 moderately severe disability (unable to attend to bodily needs without assistance and unable to walk unassisted), 5 severe disability (requires constant nursing care and attention, bedridden, and incontinent), and 6 death. Persons with a score of 0, 1, or 2 are considered to have good outcome. IVT, intravenous thrombolysis; EVT, endovascular treatment.

Comparison of characteristics between patients with good and poor outcome were provided in Table 2. After adjusting for age, NIHSS score, and baseline core volume, EVT was independently associated with good outcome [OR, 3.875; 95% Cl 1.068–14.055, *p* = 0.039] (Table 3). Reperfusion also independently predicted good outcome (OR, 7.718; 95% Cl 1.713–34.772, *p* = 0.008; Table 4). Notably, all patients with good outcome achieved recanalization at 24 h. As shown in Figure 2, EVT patients had a lower mRS score at 90-day than the untreated patients and IVT patients. In addition, patients with recanalization and reperfusion also had a lower mRS than those without.

**Table 2:** Comparison of characteristics between patients with good and poor outcome.

	**Poor outcome (*n* = 88)**	**Good outcome (*n* = 16)**	***p*-value**
Age, y	73.1 ± 11.9	68.3 ± 15.6	0.160
Female	27 (30.7)	5 (31.3)	1.000
**TREATMENT**			
IVT	69 (78.4)	7 (43.8)	0.011
EVT	19 (21.6)	9 (56.1)
**COMORBID CONDITIONS**			
Atrial fibrillation	47 (53.4)	10 (62.5)	0.827
Hypertension	63 (71.6)	13 (81.3)	0.549
Diabetes	19 (21.6)	1 (6.3)	0.298
Hyperlipidemia	24 (27.3)	5 (31.3)	0.766
Smoking	27 (30.7)	7 (43.8)	0.386
NIHSS score	16.5 (13–19)	13.5 (9–16)	0.006
Onset to imaging time, min	185 (112–237)	137 (80–219)	0.152
**RADIOLOGICAL DATA**			
Underwent CT at baseline	77 (87.5)	14 (87.5)	1.000
Baseline core volume, ml	109.9 (83.8–138.5)	101.7 (81.5–123.5)	0.454
Baseline hypoperfusion volume, ml	194.1 (151.8–252.2)	181.8 (123.9–209.4)	0.152
Baseline brain edema	0.5 (0–1)	1 (0–1)	0.621
Site of vessel occlusion
ICA	40 (45.5)	5 (31.3)	0.412
MCA	48 (54.5)	11 (68.8)
Underwent CT at 24 h^*^	50 (58.1)	9 (56.3)	1.000
24 h infarct volume, ml^*^	151.7 (84.9–251.0)	55.9 (18.4–93.5)	< 0.001
24 h brain edema^*^	3 (2–5)	1 (1–2.75)	0.001
Brain edema expansion within 24 h^*^	2 (1–3)	1 (0–1)	< 0.001
Recanalization^**^	43 (58.1)	16 (100.0)	0.001
Reperfusion^#^	23 (33.3)	12 (80.0)	0.001
HT^*^	50 (58.1)	8 (50.0)	0.591
sICH^*^	10 (11.6)	0 (0.0)	0.356

* *Analyzed in 102 patients (16 with good outcome and 86 with poor outcome)*.

** *Analyzed in 90 patients (16 with good outcome and 74 with poor outcome)*.

# *Analyzed in 84 patients (15 with good outcome and 69 with poor outcome)*.

*IVT, intravenous thrombolysis; EVT, endovascular treatment; NIHSS, National institutes of Health Stroke Scale; CTP, CT perfusion; HT, hemorrhagic transformation; sICH, symptomatic intracranial hemorrhage; mRS, modified Rankin Scale*.

**Table 3:** Multivariate regression analysis of independent predictors for good outcome (mRS < 3).

	**OR**	**95%Cl**	***p*-value**
Age	0.973	0.928–1.021	0.260
NIHSS	0.820	0.705–0.953	0.010
Baseline core volume, ml	0.998	0.983–1.021	0.838
Onset to imaging time, min	0.995	0.988–1.003	0.245
EVT (set IVT as reference)	3.875	1.068–14.055	0.039

*IVT, intravenous thrombolysis; EVT, endovascular treatment; NIHSS, National institutes of Health Stroke Scale*.

**Table 4:** Multivariate regression analysis of independent predictors for good outcome (mRS < 3).

	**OR**	**95%Cl**	***p*-value**
Age	0.944	0.891–1.000	0.052
NIHSS	0.816	0.681–0.978	0.028
Baseline core volume, ml	1.001	0.983–1.019	0.921
Onset to imaging time, min	0.996	0.986–1.005	0.354
Reperfusion	7.718	1.713–34.772	0.008

*NIHSS, National institutes of Health Stroke Scale*.

Recanalization was achieved in 21/25 (84.0%) EVT patients and 38/65 (58.5%) IVT patients. Among them, the degree of brain edema expansion was comparable between EVT and IVT groups [2 (1–3) vs. 2 (1–2) vs.); *p* = 0.144). HT rate (63.2 vs. 71.4%, *p* = 0.578) and sICH rate (7.9 vs. 9.5%, *p* = 1.000] were also comparable between IVT and EVT groups. In patients achieved recanalization, EVT group have higher reperfusion rate [97.1% (82.1–99.6%) vs. 78.4% (42.2–95.1%), *p* = 0.021] compared with IVT group.

**Figure 3:**
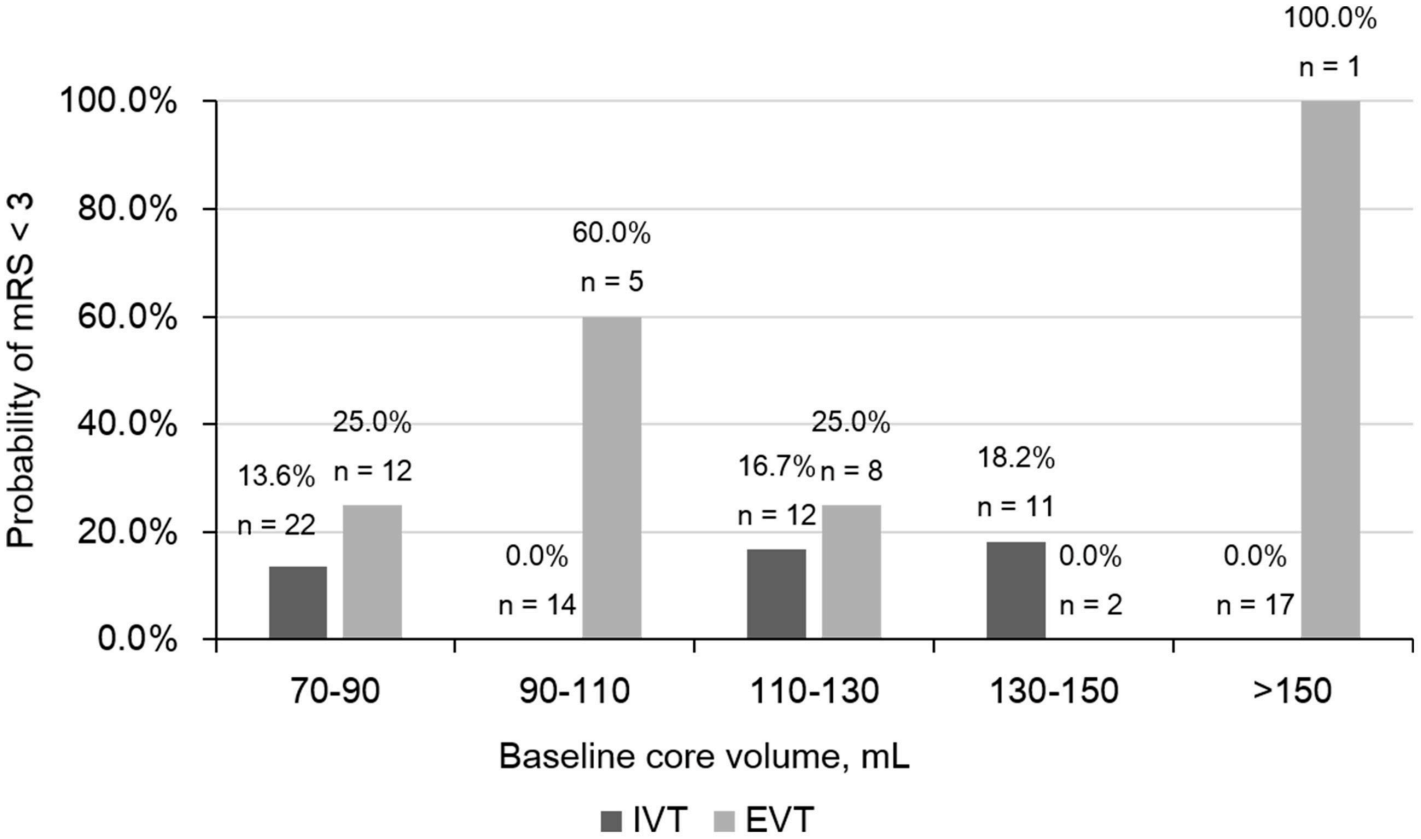
Comparison of Probability of modified Rankin scale (mRS) < 3 in different baseline core volume subgroups. IVT, intravenous thrombolysis; EVT, endovascular treatment.

The relationship between baseline core volume and probability of good outcome was shown in Figure 3. We didn't find out a cut-off for baseline core volume to select patients for reperfusion therapy.

## Discussion

Our study revealed that, among eligible AIS patients with large core (≥70 ml), EVT may still increase the rate of favorable outcome compared IVT. Reperfusion were also associated with good outcome. EVT didn't increase the rate of sICH. Our results suggested that patients with large core might still benefit from EVT.

Our finding is slightly different from previous studies which demonstrated the clinical benefit of successful recanalization or reperfusion in patients with pretreatment DWI volume ≥70 ml. Tisserand et al. found that in patients treated by IVT, recanalizers experienced an increased rate of favorable outcome compared with non-recanalizers (36 vs. 10%) (13). Gilgen et al. reported that in patients who underwent EVT, reperfusers experienced an increased rate of favorable outcome compared with non-reperfusers (33 vs. 8%) (10). Olivot et al. also observed that in EVT patients, reperfusers were more likely to achieve favorable outcome compared to non-reperfusers (42.9 vs. 8.3%) (4). Two other studies used DWI-Alberta Stroke Program Early CT score (ASPECTS) to define large infarct also demonstrated that recanalizers were more likely to achieve favorable outcome than non-recanalizers in IVT and EVT treated patients, respectively (14, 15). To summarize, these studies proved that recanalization and reperfusion can improve clinical outcome for patients with core volume ≥70 ml treated with either IVT or EVT, but our study directly compared the outcome between EVT and IVT, and proved that patients with core volume ≥70 ml could benefit from EVT compared to IVT treatment.

As widely recognized, compared to IVT, EVT significantly increases the rate of recanalization and reperfusion, which was also demonstrated in our study among patients with core volume ≥70 ml, which may explain the relatively low rate of good outcome in IVT patients. On the other hand, pre-clinical study found that significant thrombus reduction could not be observed until 1 h after the initiation of thrombolysis (16), which indicated the relatively delayed recanalization in IVT patients compared with EVT, resulting in further penumbra loss. Recent studies confirmed that imaging to reperfusion interval was negatively associated with favorable outcome (17, 18). Therefore, the unsatisfied outcome in IVT-treated patients may be related to longer imaging to reperfusion time compared to EVT patients, since the time from onset to imaging was similar in two groups. However, since we could not calculate the actual imaging to reperfusion interval in IVT group, this hypothesis still requires further confirmation.

Core ≥70 ml was considered to be an exclusion criteria for reperfusion therapy in most clinical trials. The concept of malignant profile was first brought up by Diffusion and Perfusion Imaging Evaluation for Understanding Stroke Evolution (DEFUSE) investigators. They found that among 6 patients with DWI volume >100 ml, all 3 patients who had early reperfusion had a sICH and all 3 died. It was confirmed again in DEFUSE and EPITHET database. Among 27 patients with malignant profile (defined by Tmax >8s) who underwent IVT, patients achieving reperfusion were more likely to have poor outcome compared with non-reperfusers due to increased rate of parenchymal hematoma (19). And another group also found that patients with baseline DWI lesion > 65 ml had a very high rate of poor outcome, regardless of IVT or not (3). As a result, core >70 ml was considered to be a relative contraindication for reperfusion therapy, and patients with this profile were ruled out for the promising EVT clinical trials. However, the sample size of these 3 studies were too small to draw a strong conclusion. There was also a similar study about EVT in 2009 which presented that all 6 patients with initial infarcts ≥70 ml had poor outcome despite of 50% recanalization rate after EVT (2). However, the thrombectomy devices used in this study before 2009 were not quite developed. In our study, we revealed that EVT did not increase the risk of sICH, though it was associated with HT, indicating that the safety of EVT is not less than IVT in these patients. Besides, in patients who achieved recanalization, the risk of HT, sICH and the degree of edema expansion between IVT and EVT were all comparable, indicating that reperfusion injury caused by recanalization was not different between IVT and EVT.

Our results suggested that EVT may still be considered in patients with large core (>70 ml) as they may benefit from EVT. We are clear that core volume strongly affects outcome, and we also believe that there may be a cut-off volume for EVT exclusion, but from the current study we are convinced that 70 ml is not the point. The withdrawal of patients with core >70 ml from the thrombectomy study was largely due to the futile reperfusion revealed in IVT studies, but in fact, the benefit from the shortening of reperfusion time and higher reperfusion rate in EVT still existed. Besides, in the latest 2018 Guidelines for the Early Management of Patients With Acute Ischemic Stroke, Multimodal CT and MRI are not recommended for patients within 6 h of stroke onset (20), based on the results of THRACE (21) and MR CLEAN (22) trials, which only used baseline NCCT for triage of thrombectomy. Our results may provide evidences that core volume is not necessary for selecting thrombectomy-eligible patients within 6 h, though further studies are needed to determine the usage of advanced imaging paradigms.

Our study has several limitations. The first shortcoming is related to its moderate sample size, especially the EVT group, but it also had the largest sample compared to the other studies focus on patients with core ≥70 ml. Our results remain to be confirmed in larger multi-center trials. Secondly, in our study, we analyzed both CT and MR images, which raised an unavoidable question about the accuracy of CTP-assessed infarct core. Demeestere et al. demonstrated that the accuracy of CTP core volume to predict large DWI lesion was excellent (23), and trials like EXTEND-IA and DEFUSE 3 also included both CTP and MRP assessed patients (24). Besides, since in our study, the percentage of patients underwent CTP were comparable in different treatment groups and different outcome groups, the heterogeneity between CT and MR imaging might not affect our main results.

In conclusion, our data indicate that patients with large core should still not be strictly excluded from EVT, as favorable outcome was achieved in 31% patient with core volumes ≥ 70 ml after EVT.

## Author contributions

ZC and RZ drafted and revised the manuscript, participated in study concept and design, conducted the statistical analyses, analyzed, and interpreted the data. ML participated in study concept and design, data interpretation, and made a major contribution in revising the manuscript. YZ and XY assisted in data collecting. XG, FS, and MZ participated in the study design and made contribution in revising the manuscript.

### Conflict of interest statement

The authors declare that the research was conducted in the absence of any commercial or financial relationships that could be construed as a potential conflict of interest.
